# Our Contemporaries

**Published:** 1915-08

**Authors:** 


### OUR CONTEMPORARIES.

The Wisconsin Medical Recorder was merged, July 1, with the Physicians' Drug News of Newark, N. J.

